# A comparative analysis of NLRP3-related inflammatory mediators in synovial fluid in temporomandibular joint osteoarthritis and internal derangement

**DOI:** 10.1186/s12891-021-04092-0

**Published:** 2021-02-26

**Authors:** Mengying Jia, Yaoguang Lv, Yingjie Xu, Zhongcheng Gong

**Affiliations:** 1 Oncology Department of Oral and Maxillofacial Surgery of The First Affiliated Hospital of Xinjiang Medical University, Urumqi, 830054 Xin Jiang Province China; 2 The Stomatology College of Xinjiang Medical University, Urumqi, 830054 Xin Jiang Province China

**Keywords:** Condylar degeneration, Temporomandibular joint osteoarthritis, NLRP3, Hyaluronic acid

## Abstract

**Background:**

The nucleotide-binding oligomerization domain-like receptor pyrin domain containing 3 (NLRP3) inflammasome signaling pathway is a highlighted topic in the field of inflammation. However, there is little research on the relationship between the NLRP3 inflammasome pathway and temporomandibular joint osteoarthritis (TMJOA). The aim of this study was to examine the expression of inflammatory mediators related to the NLRP3 inflammasome in the synovial fluid of patients with condylar cartilage degeneration and verify the clinical effects of sodium hyaluronic acid (HA) treatment on TMJOA.

**Methods:**

Patients diagnosed with temporomandibular joint internal derangement (TMJID) without condylar defects and TMJOA with condylar defects were divided into two groups. There were thirty patients in each group, and inflammatory mediators related to the NLRP3 inflammasome, including interleukin-1 beta (IL-1β), IL-18, NLRP3, and cysteinyl aspartate specific proteinase 1 (CASP1), in synovial fluid were measured by enzyme-linked immunosorbent assay (ELISA). Eighteen patients in the TMJOA group were retested after two HA treatments to evaluate the therapeutic effects of HA.

**Results:**

IL-1β, IL-18, NLRP3 and CASP1 were all positive in the two groups, and TMJOA patients with condylar defects had higher expression of these molecules than TMJID patients (*P* < 0.05). IL-1β, IL-18, and NLRP3 were decreased after two HA treatments (*P*<0.05), but there was no significant difference in CASP1 after two HA injections (*P* = 0.549).

**Conclusions:**

The NLRP3 inflammasome signaling pathway may be involved in condylar degeneration. HA could reduce some inflammatory molecules to alleviate inflammation.

**Supplementary Information:**

The online version contains supplementary material available at 10.1186/s12891-021-04092-0.

## Background

Temporomandibular joint osteoarthritis (TMJOA) is a dysfunction characterized by articular disc-condylar disorder, cartilage damage and extracellular matrix degeneration. Condylar degeneration is the direct result of TMJOA progression and threatens quality of life because of high occurrence and serious clinical symptoms [1]. Due to the low blood and lymph supply in articular cartilage tissue, self-repair is unsuccessful once progressive destruction of condylar cartilage has begun [2, 3]. Despite various medicines, surgery and tissue engineering techniques, the clinical therapeutic effects are not satisfactory. Presently, the mechanism of cartilage degeneration remains unclear, and it is pivotal to identify novel biological markers to assess the severity of local TMJOA and develop an optimal treatment. The risk factors for OA include factors related to individual susceptibility, as well as those affecting biomechanical joint stability. Previous studies have shown that malocclusion is an independent risk factor for temporomandibular joint disorder (TMD), which leads to abnormal mechanical stimulation of the condyle via periodontal proprioceptive mechanisms [4, 5]. OA has different etiologies but the same inflammatory pathology, and most inflammatory pathways promote the progression of OA [6, 7].

**Table 1:** Clinical characteristics with TMJID and TMJOA patients(*n* = 60)

	TMJID	TMJOA
Gender (n)	Female/18	Female/20
	Male/12	Male/10
Age (year)	29.48 ± 6.95	37.72 ± 8.89
Crossbite (n)	3	5
Scissors-bite (n)	4	7
Overbite (n)	6	7
Other types of malocclusion (n)	17	11
Joint pain (n)	7	6
Limited mouth opening (n)	9	11
Asymmetrical face (n)	1	4

**Table 2:** Mouth opening and visual analogue scale (VAS) in TMJOA group with twice HA treatments (*n* = 18)

	First injection	Second injection	*P*
Mouth opening (cm)	3.37 ± 0.96	3.87 ± 0.075	< 0.001
VAS	1.43 ± 0.92	0.25 ± 0.44	< 0.001

**Table 3:** Comparison concentration of IL-1β, IL-18, NLRP3 and CASP1 between TMJID group and TMJOA group

	TMJID	TMJOA	*t*	*P*
IL-1β (pg/ml)	358.62 ± 148.20	533.58 ± 329.73	2.65	0.011
IL-18 (pg/ml)	260.14 ± 149.93	347.08 ± 85.49	2.76	0.008
NLRP3 (ng/ml)	0.46 ± 0.45	0.58 ± 0.1	5.73	< 0.001
CASP1 (pmol/L)	157.4 ± 32.97	179.91 ± 37.97	2.45	0.017

**Table 4:** Changes of IL-1β, IL-18, NLRP3 and CASP1 concentration in TMJOA groups with twice HA injections

	First injection	Second injection	*P*
IL-1β (pg/ml)	656.50 ± 272.06	267.15 ± 159.61	< 0.001
IL-18 (pg/ml)	397.73 ± 60.03	132.06 ± 101.24	< 0.001
NLRP3 (ng/ml)	0.61 ± 0.11	0.42 ± 0.02	< 0.001
CASP1(pmol/L)	165.34 ± 29.48	169.47 ± 37.72	0.549

**Fig. 1:**
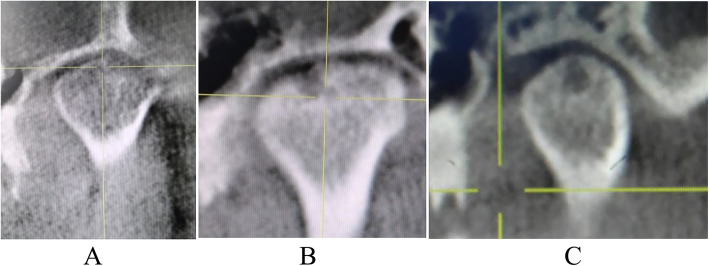
CBCT manifestations of TMJOA patients. **a**: TMJOA with condylar bone defect of surface; **b**: TMJOA with obvious condylar defect; **c**: Cystic degeneration in coronal image

**Fig. 2:**
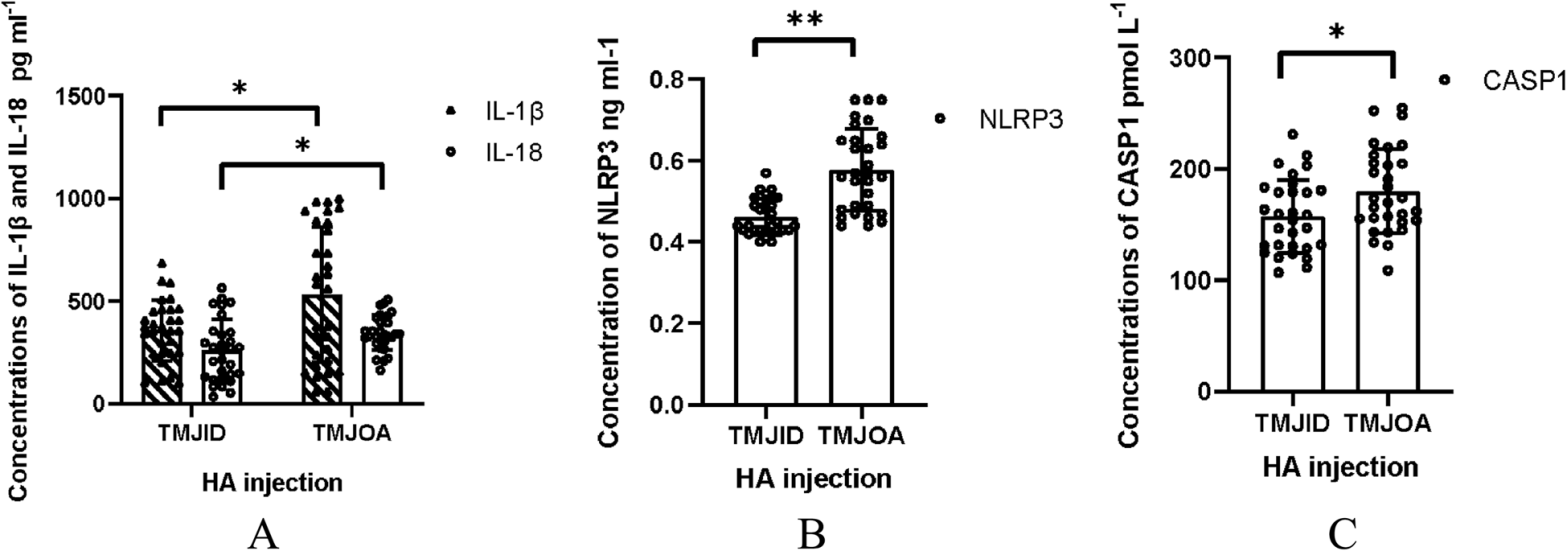
Comparison concentrations of IL-1β, IL-18, NLRP3 and CASP1 between TMJID group and TMJOA group. **a**: Concentrations of IL-1β, IL-18; **b**: Concentration of NLRP3; **c**: Concentration of CASP1. *: *P* < 0.05, **: *P* < 0.001

**Fig. 3:**
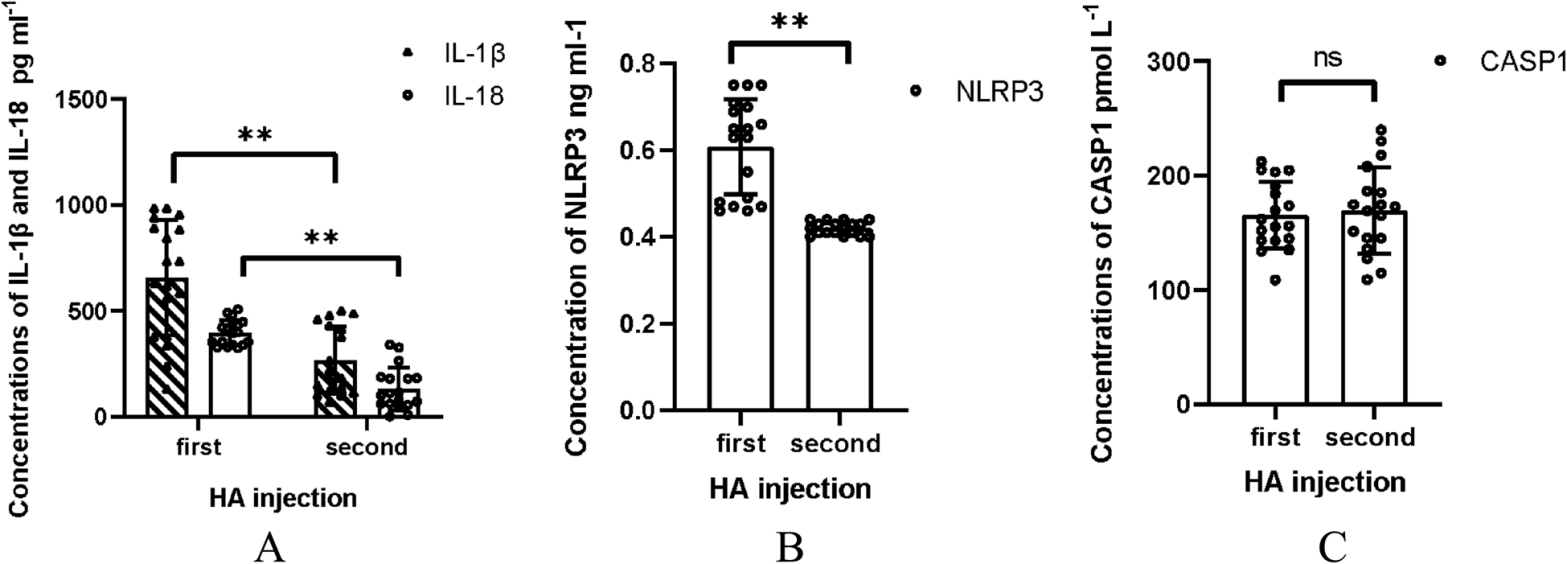
Concentration changes of IL-1β, IL-18, NLRP3 and CASP1 with twice HA intra-articular injections. **a**: Concentrations of IL-1β, IL-18; **b**: Concentration of NLRP3; **c**: Concentration of CASP1. *: *P* < 0.05, **: *P* < 0.001

Inflammatory cytokines, including interleukin-1 beta (IL-1β) and IL-18, are crucial in the pathogenesis of OA. IL-1β can induce thermal and mechanical hyperalgesia via activated transient receptor proteins (TRPs). IL-18 has been demonstrated to trigger cartilage proteoglycan degradation. The nucleotide-binding oligomerization domain-like receptor pyrin domain containing 3 (NLRP3) inflammasome has been demonstrated to be an important platform responsible for the secretion of mature IL-1β and IL-18. Recent studies have shown that activation of the NLRP3 inflammasome participates in various types of inflammation and a new programmed cell death named pyroptosis. Activation of the NLRP3 inflammasome requires a diverse range of stimuli, including pathogen-associated molecular patterns (PAMPs) and damage-associated molecular patterns (DAMPs) [8, 9]. After activation, intracellular NLRP3 engages pro-cysteinyl aspartate specific proteinase 1 (pro-CASP1) through apoptosis-associated speck-like protein containing a CARD (ASC), and activated CASP-1 catalyzes proteolytic cleavage of the precursors of IL-1β and IL-18 to generate their mature forms and initiates inflammation [10]. However, there have been few reports on the relationship between the NLRP3 inflammasome pathway and TMJOA, and we hypothesize that inflammatory mediators related to the NLRP3 inflammasome contribute to the pathogenesis of TMJOA. Therefore, we compared the expression of IL-1β, IL-18, NLRP3 and CASP1 in synovial fluid from temporomandibular joint internal derangement (TMJID) patients and TMJOA patients to verify the activation of the NLRP3 inflammasome in condylar degeneration. Moreover, we also measured the same molecules after two hyaluronic acid (HA) therapeutic treatments in the TMJOA group to evaluate the therapeutic effect of HA.

## Methods

### Objects of study

The Ethics Committee of The First Affiliated Dental Hospital of Xinjiang Medical University approved this study (number K201910–04). All patients were from The First Affiliated Hospital of Xinjiang Medical University and were diagnosed with TMD by the same expert in oral and maxillary surgery from October 2018 to April 2019. According to the clinical symptoms and cone-beam computed tomography (CBCT) or magnetic resonance imaging (MRI) examination, patients were screened and separated into the TMJID group without condylar bone defects or the TMJOA group with condylar bone defects, and there were 30 patients in each group. Informed consent was obtained from all patients. The inclusion criteria were as follows: (1) Based on the research diagnostic criteria of temporomandibular joint disorder (RDC/TMD), TMJID patients exhibited TMJ disc displacement with reduction and TMJ disc displacement without reduction. TMJOA patients presented with TMJ clicking and condylar degeneration. (2) Patients over 18 years old presented with oral malocclusion. The exclusion criteria were as follows: (1) Patients had mental illness. (2) Patients had habit of unilateral mastication. (3) Patients had history of TMJ trauma, autoimmune disease, rheumatoid arthritis, or acute or chronic infections. (4) Patients had serious cardiovascular, liver, kidney or other endocrine diseases.

### Groups of study

The subjects were divided into two groups (30 patients per group): the TMJID group without condylar bone defect was confirmed according to the RDC/TMD [11], and CBCT or MRI examination revealed no condylar bone destruction, while the TMJOA group with condylar bone defect were exhibited linear shadows, cysts or osteophyte formation in axial and coronal images with CBCT (Fig. 1) or MRI examination and did not exhibit symptoms of internal derangement in the TMJ. To evaluate the therapeutic effect of HA, 18 out of 30 TMJOA patients who received two HA intra-articular injections once per week for 2 weeks were included in the self-control group.

### Clinical data records

Clinical data were recorded by two professional doctors for consistency, and these parameters included sex, age, symptoms of the temporomandibular joint, types of malocclusion upon oral examination, visual analog scale (VAS) score, mouth opening and imaging manifestations. The follow-up information was recorded in detail to evaluate clinical outcomes.

### Collection of synovial fluid and HA intra-articular injection

Synovial fluid was collected at the time of HA intra-articular injection. The HA was produced by Kunming Baker Nortion Pharmaceutical Sales and had an average molecular weight of 800–1200 kDa. Patients were instructed on the importance of treatment and informed consent was freely provided. After head deviation on the seat, 3 ml (mL) of lidocaine was injected into the lesion in the anterior 1-cm (cm) of the tragus. The needle tip moved forward obliquely, up and inside, and retreated approximately 1 mL from the posterior oblique surface of the condylar to flush the immune substances in the articular cavity, and 1.5 mL of synovial fluid was collected. To treat TMD, 1 mL of HA was injected in the same site, and the injection was performed once per week for 2 weeks. The synovial fluid was centrifuged in a timely manner (12,000 r/min, 15 min, 4 °C) and frozen at − 80 °C.

### Enzyme-linked immunosorbent assay (ELISA)

IL-1β (Wuhan Boster; China; EK0392-96 T), IL-18 (Wuhan Boster; China; EK086), NLRP3 (Shanghai Jonln; China; JL18318), and CASP1 (Shanghai Jonln; China; JL13683) were analyzed according to the instructions of the highly sensitive and specific ELISA kit. The absorption was measured at 450 nm on a Thermo Scientific microplate reader.

The assay can determine the levels in unknown samples according to the standard curve, which is made based on the absorption values of the standard sample.

### Statistical analysis

The SPSS 21.0 software package was used for statistical analysis and recording the data. Values are presented as the mean and standard deviation (SD). The Shapiro-Wilks test was used to assess the normality and data distribution, and the homogeneity of variance was analyzed with Leven’s test. Statistical significance was assessed by Student’s t-test if the homogeneity of variance was equal or evaluated by Satterthwaite’s test if the homogeneity of variance was not consistent for the two compared groups. Data were analyzed with paired t-tests before and after the two treatments. There was a significant difference between the two groups or the self-control group if *P*<0.05.

## Results

### Clinical characteristics of the two groups

The clinical characteristics of the two groups were summarized (Table 1). The TMJID group included 18 females and 12 males, 3 patients with crossbite, 4 patients with scissors-bite, and 6 patients with overbite upon oral examination. The TMJOA group included 20 females and 10 males, 5 patients with crossbite, 7 patients with scissors-bite, and 7 patients with overbite upon oral examination. There was no significant difference in sex or age composition between the two groups (*P* < 0.05), and elderly females accounted for a larger proportion in the two groups. Patients in the two groups had different levels of TMD dysfunction, including joint pain, limited mouth opening and asymmetrical faces.

### Clinical effects of two HA injections on the TMJOA group

TMD was alleviated after the second HA treatment. There was increased mouth opening and decreased VAS scores in TMJOA patients after two HA injections (*P* < 0.001) (Table 2).

### The concentrations of IL-1β, IL-18, NLRP3 and CASP1

Positive expression of IL-1β, IL-18, NLRP3, and CASP1 was detected in the two groups. However, there were higher concentrations of these molecules in the TMJOA group than in the TMJID group (*P* < 0.05) (Table 3, Fig. 2).

### The therapeutic effect of HA on IL-1β, IL-18, NLRP3 and CASP1

We analyzed the therapeutic effects of HA injection between the first and second HA treatments and observed the greatest reductions in the concentrations of IL-1β, IL-18, and NLRP3 (*P* < 0.001) (Table 4, Fig. 3). These decreased inflammatory factors were consistent with the alleviation of clinical joint symptoms. However, there was no significant difference in CASP1 after two HA injections (*P* = 0.549) (Table 4, Fig. 3).

## Discussion

TMJOA is a degenerative joint disorder that occurs predominantly in elderly individuals and women, and complex factors lead to similar pathological changes in joint destruction, synovitis, and clinical signs of joint pain and articular dysfunction. In our study, there was no significant difference in age or sex composition between the TMJID group and TMJOA group, and the proportion of elderly females was increased. Condyle cartilage defects rarely self-heal, which is related to inflammatory pathology. The NLRP3 inflammasome participates in multiple inflammatory and immune responses; thus, we hypothesized that the NLRP3 inflammasome pathway might be present in TMJOA. Our findings suggested that IL-1β, IL-18, NLRP3 and CASP1 were highly expressed in degenerative TMJOA synovial fluid, which was consistent with NLRP3 inflammasome pathway involvement. Thus, the NLRP3 inflammasome pathway may participate in condylar degeneration. Our data also indicated that HA protected condylar tissue from progressive degradation by alleviating clinical symptoms and decreasing the expression of some inflammatory molecules. These results provide a basis for further study of the mechanism of TMJOA.

Inflammation is a complex biological response of tissues to harmful factors and is accompanied by the release of inflammatory mediators. Previous studies have shown that the expression of inflammatory mediators, including IL, matrix metalloproteinase (MMP) and prostaglandin E2 (PGE2), in the synovial fluid of OA patients is higher than that in normal controls [12, 13]. In addition, inflammation also dysregulates the catabolism of the cell matrix and leads to the deterioration of the internal environment. Osseous changes generally occur in response to TMJ inflammation, while CBCT images clearly show hard tissues [14, 15]. Our study used groupings according to CBCT or MRI to accurately distinguish condylar destruction.

IL-1β and IL-18 have important functions in mediating innate and adaptive immunity, mainly through the induction of cyclooxygenase type-2, MMP and the hydrolytic enzyme aggrecan to suppress the biosynthesis of extracellular matrix, leading to the recruitment of neutrophils to sites of inflammation and inducing cell death. Both IL-1β and IL-18 share similar proinflammatory properties and are produced as biologically inactive pro-forms [16–18]. Activated CASP-1 is required for proteolytic processing and the release of the mature forms of IL-1β and IL-18, which are activated following inflammasome formation, especially the NLRP3 inflammasome [19]. In our study, despite the low positive concentration of NLRP3 in the two groups, the higher concentrations of IL-1β, IL-18, NLRP3 and CASP1 in TMJOA patients than in TMJID patients suggested the possibility of NLRP3 inflammasome activation.

The NLRP3 inflammasome is the best characterized in the context of pyroptosis, which exhibits cell swelling and lysis. Canonical NLRP3 inflammasome activation begins with a priming signal induced by nuclear factor kappa B (NF-κB) signaling, which upregulates the transcription of NLRP3 and pro-IL-1β. An activation signal is then initiated by DAMPs and PAMPs, which induce inflammasome assembly by the recruitment of ASC and caspase-1. DAMPs are endogenous molecular structures, including high-mobility group box 1 (HMGB1), IL-13, heat-shock proteins (HSPs), S100 proteins, ATP, and mitochondrial DNA, that are released into the extracellular environment through tissue injury or hypoxia stimulation and participate in the canonical NLRP3 inflammasome pathway that is controlled by CASP1 [20]. PAMPs are molecular structures that are found in microbes, including lipopolysaccharide (LPS), which can participate in canonical and noncanonical NLRP3 pathways and pyroptosis controlled by CASP1 or CASP-4/5/11 [21]. The TMJ is a rotating-hinge joint that is suitable for occlusal mechanical environments. In general, there are no bacteria in the closed joint space, and the temporomandibular joint accepts biomechanical stimulation, such as fluid shear stress transmitted by abnormal occlusal contact. This stress may induce mitochondrial dysfunction [22], changes in calcium (Ca^2+^) flux and endoplasmic reticulum stress [23], and we believed that DAMPs were the main stimuli. In this study, malocclusion was most likely a source of DAMPs because all patients in both groups had oral malocclusion.

To date, a limited number of studies have described the role of the NLRP3 inflammasome in the pathogenesis of OA. The associations of NLRP3 with OA risk factors, cartilage degeneration, synovitis and OA pain [24] are depicted in detail. Patients with OA risk factors (such as metabolic disorders, aging, infectious joint diseases and injuries) can produce a variety of DAMPs, including adipokines, microcrystalline and uric acid. Microcrystals are regarded as DAMPs and have been found in the knee joint of OA patients [25]. These crystals induce OA by stimulating the release of IL-1β from macrophages and activating NLRP3 in a process that is dependent on K+ efflux and reactive oxygen species (ROS) [26]. In addition, released IL-1β and IL-18 react on the surface of chondrocytes and induce further cartilage degeneration. Synovitis-associated inflammation is a known cause of cartilage degradation. Studies have shown that the expression of NLRP3 in OA synovial tissue is five times higher than that in normal tissue, and in the context of cartilage degeneration, IL-1β is more likely to be produced by synovial tissue, especially synovial macrophages, by DAMP-mediated activation NLRP3, which releases IL-1β into the synovial fluid to amplify inflammation in cartilage [27]. On the other hand, upregulated proinflammatory mediators are associated with OA pain, and IL-1β and IL-18 can increase nociceptive input in joint tissue, which is called hyperalgesia [28].

Additionally, we evaluated the therapeutic effect of two HA injections on NLRP3 inflammatory molecules. The application of HA by intra-articular injection plays an important role in the protection, nutrition and function of joints. HA also regulates electrolytes and water in extracellular fluid, lubricates joints, and resists infection [29]. Our current study also verified the anti-inflammatory effect of HA, as indicated by the alleviation of symptoms and decreased concentrations of IL-1β, IL-18, and NLRP3. However, there was no statistical significance in the concentration of CASP1 between the two HA injections, which may be because the short follow-up time did not allow for the establishment of a complete immune response. Of course, there is a significant amount of literature that states that HA can also induce inflammation within joints. The possible reasons include bacterial infection caused by nonstandard clinical operations, failure to completely withdraw joint effusion before sodium hyaluronate injection, and individual intolerance. We also need to perform standard injections and expand the sample size to evaluate the anti-inflammatory effect of HA in the future. In addition, the limitation of this study is that the sample size was small, and the grouping was rough. We expect to enlarge the sample size and group samples based on the pathological stage of OA in future studies to verify the relationship between NLRP3 inflammatory mediators and OA. In addition, histopathological studies of temporomandibular joint specimens are needed.

Currently, targeted drug therapy tends to be well established. Inhibition therapy targeting the abovementioned inflammatory molecules may become a new strategy for progressive osteoarthritis when mechanical stress cannot be immediately alleviated, which also makes individualized treatment of articular joint destruction possible.

In conclusion, inflammatory mediator-related NLRP3 inflammasomes were highly expressed in OA patients. Activation of the NLRP3 inflammasome in chondrocytes or synovial cells might be a new reason for cartilage degradation, and intra-articular injection of HA was effective in alleviating clinical symptoms and decreasing some inflammatory factors.

## Supplementary Information

**Additional file 1.**

**Additional file 2.**

## Data Availability

The datasets used and/or analyzed during the current study are available from the corresponding author on reasonable request.

## Abbreviations

TMJOA: Temporomandibular joint osteoarthritis
HA: Temporomandibular joint osteoarthritis
TMJID: Temporomandibular joint internal derangement
IL-1β: Interleukin-1beta
IL-18: Interleukin 18
NLRP3: Nucleotide-binding oligomerization domain-like receptor pyrin domain containing 3
CASP1: Cysteinyl aspartate specific proteinase 1
PAMPs: Pathogen-associated molecular patterns
DAMPs: Damage-associated molecular patterns
Elisa: Enzyme-linked immunosorbent assay
CBCT: Cone-beam computed tomography
MRI: Magnetic resonance imaging
MMP: Metalloproteinase
PGE2: Prostaglandin E2
LPS: Lipopolysaccharide
SD: Sprague-dawley
